# Age‐Related Complement C3 Drives Memory Impairments and Associated Neuropathologies in a Mouse Model

**DOI:** 10.1111/acel.70145

**Published:** 2025-06-20

**Authors:** Miaomiao Du, Yujia Wang, Xinyuan Wang, Yifei Liu, Fuchuan Xiao, Jing Zhang, Xia Meng, Kunhe Ma, Na Wu, Baian Chen, Jing Lu

**Affiliations:** ^1^ Department of Laboratory Animal Sciences, School of Basic Medical Sciences Capital Medical University Beijing People's Republic of China; ^2^ Laboratory Animal Resource Center Capital Medical University Beijing People's Republic of China; ^3^ Center of Alzheimer's Disease, Beijing Institute of Brain Disorders Capital Medical University Beijing People's Republic of China

**Keywords:** aging, complement C3, complement C3 targeting, learning and memory

## Abstract

Aging is the greatest risk factor for learning and memory disorders; dementia prevalence significantly increases with age due to numerous molecular changes in the body. Although research has consistently shown that aging leads to learning and memory impairments, the molecular mechanisms linking aging to these cognitive deficits remain incompletely understood. Previous studies have revealed that complement C3 levels increase with age in humans, monkeys, and mice; elevated C3 expression is also observed in the brains of dementia patients. These data suggest that C3 plays critical roles in initiating learning and memory impairments. To investigate whether C3 contributes to these deficits during aging, we developed a transgenic mouse model with elevated C3 expression to simulate age‐related increases. Mice with increased C3 expression showed impaired learning and memory, along with synaptic loss, neuronal loss, and astrocytosis. Quantitative polymerase chain reaction microarray and cellular assays revealed that C3 elevation may impair cognitive functions by affecting insulin signaling pathways. Notably, antibody therapy targeting complement C3 in SAMP8 mice, which naturally exhibit increased brain C3 levels, alleviated their learning and memory deficits. These findings suggest that age‐related complement C3 elevation drives memory impairments and associated neuropathologies; targeting complement C3 may alleviate these deficits.

## Graphical abstract

Age‐related complement C3 elevation induces memory impairments and neuropathology by dysregulating astrocytic insulin signaling and triggering mitochondrial dysfunction. Critically, antibody blockade of C3 in mouse brain tissue effectively reversed learning and memory deficits in mice, demonstrating that targeting C3 is a promising therapeutic strategy.

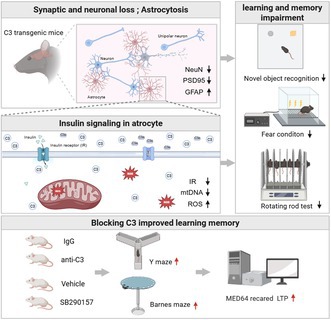

**Abbreviations**
AD: Alzheimer's disease
Anti‐C3 mice: mice after cerebral injection of complement C3 antibody
C3^+/+^ mice: transgenic mice with elevated C3 expression
C3aR: C3a receptor
CD11b: alpha chain of complement receptor 3
DI: discrimination index
IgG mice: mice after cerebral injection of IgG
IRS2: insulin receptor substrate 2
mtDNA: mitochondrial DNA
OPLS‐DA: orthogonal partial least squares discriminant analysis
ROS: reactive oxygen species
SB290157: C3aR competitive antagonists
TEM: employed transmission electron microscopy

## 1 Introduction

Aging constitutes the gradual decline in physiological functions and structural integrity of an organism over time (Rose 2009). Experts estimate that by the mid‐21st century, the global population of people aged 60 and older will reach 2.1 billion, highlighting a substantial increase in population aging (Wu et al. 2022). Concurrently, the prevalence of dementia among older people is rising, posing a serious threat to physical health and quality of daily life.

Age is recognized as a primary risk factor for learning and memory impairments (Wyss‐Coray 2016). Cognitive abilities progressively decline with age. Neurodegenerative diseases such as dementia—especially Alzheimer's disease (AD)—are strongly linked to aging; incidence rates significantly increase with age (Wyss‐Coray 2016). Numerous molecular changes occur during aging, potentially affecting DNA integrity, mitochondrial function, immune system dynamics, nutrient metabolism, and histone stability (Hou et al. 2019). Despite the roles of various molecules in age‐related cognitive decline, aging is a highly complex process; current research has not fully elucidated the molecular relationships between aging and learning and memory impairments. Considering that immune system function alters with age, there is a need to explore the connections of complement C3 in the innate immune system with age‐related learning and memory impairments.

Complement C3 is a serum protein essential to the human immune system. Produced in the liver, it is a core component of the complement system, which helps protect the host from infections, clear cellular debris, and remove misfolded proteins; all of these processes are critical for maintaining brain homeostasis (Stephan et al. 2012). C3 is widely expressed in the brain, including in regions associated with learning and memory, such as the hippocampus, olfactory cortex, and temporal lobe. In addition to its immune role, C3 may influence cognitive function by affecting neurogenesis and synaptic plasticity. Brain C3 levels are closely linked to learning and memory functions. Studies indicate that C3 and C3a receptor (C3aR) deficiencies impact adult hippocampal neuron survival and morphology, as well as pattern separation and cognitive flexibility (Westacott et al. 2021). C3 expression in specific brain regions may increase during development, particularly in areas with high synaptic activity. C3 can be cleaved into C3a and C3b through the complement activation pathway. C3b binds to receptors on the cell membrane, such as CR3 (CD11b/CD18), through interactions that may modulate microglial phagocytic activity and support synaptic pruning (Luchena et al. 2018). In neurodegenerative diseases such as AD, synaptic pruning may be overactive, leading to excessive synaptic loss (Schafer et al. 2012). Additionally, there is evidence that C3‐deficient mice exhibit reversals in age‐related neuronal damage and cognitive impairment (Shi et al. 2015).

An intriguing phenomenon is the age‐related increase in complement C3 expression. A gene chip study comparing young (20–59 years) and older (60–99 years) populations revealed significant upregulation of the C3 gene in the hippocampus, frontal gyrus, and central posterior gyrus (Cribbs et al. 2012). Research has shown significant increases in C3d levels in the white and gray matter of older rhesus monkeys, as well as in aged C57 mice (Duce et al. 2006; Shi et al. 2015; Wu et al. 2022). Similar findings were observed in dementia patients, where C3 expression was elevated in brain tissue and cerebrospinal fluid; C3b levels in the brains of asymptomatic frontotemporal dementia carriers showed an inverse correlation with frontal lobe volume (van der Ende et al. 2022; Yasojima et al. 1999). These findings indicate a consistent upregulation of complement C3 with advancing age in human populations, monkeys, and mice, as well as in the brains of dementia patients.

Previous studies have largely focused on C3‐deficient mice to investigate the relationships of complement activity with synaptic pruning, learning, and memory. However, it remains unclear whether complement C3 serves as a core molecule that drives learning and memory impairments and what molecular mechanisms might be involved. Current interventions for cognitive impairment are suboptimal, with limited precision in available treatments and frequent adverse effects associated with therapeutic drugs. This study investigated whether increased expression of complement C3 induces learning and memory impairments by analyzing C3 transgenic mice with elevated C3 levels and exploring possible molecular mechanisms. These findings will contribute to a foundational understanding of the formation, progression, and prevention of cognitive impairments in related diseases.

## 2 Materials and Methods

### 2.1 Mouse Handing

Using CRISPR/Cas9 technology, the mouse C3 gene (NCBI Reference Sequence: NM_009778.3) was inserted into the ROSA26 locus (NCBI Reference Sequence: NR_027008.1) on chromosome 6 in C57BL/6J mice to generate C3 knock‐in mice. The CAG promoter‐mouse C3cDNA‐rBGpA cassette was cloned into intron 1 of ROSA26. Cas9 and gRNA were co‐injected with the targeting vector into fertilized eggs to produce positive F0 mice. Mice were genotyped by polymerase chain reaction (PCR) using the following primers:

Forward 1: 5′‐AAA GAT CGC TCT CCA CGC CCT AG‐3′

Reverse 1: 5′‐GAT GGG GAG AGT GAA GCA GAA CG‐3′

Forward 2: 5′‐GCA TCT GAC TTC TGG CTA ATA AAG‐3′

Reverse 2: 5′‐ATG GGA AGT TAG TAG CAA ACA AGA G‐3′

The knock‐in mice were generated by Cyagen Biosciences (Suzhou, China) and housed in a barrier environment at the Experimental Animal Facility of Capital Medical University, China. A total of C3 transgenic mice (30 males and 30 females) were used in this study. A total of 4‐month‐old SPF SAMP8 mice (30 males) and SAMR1 mice (10 males) were purchased from Department of Laboratory Animal Science, Peking University Health Science Center. Environmental conditions were maintained at 21°C ± 2°C with 40%–70% humidity, a 12‐h light/dark cycle, and free access to food and water. All experiments were performed in accordance with protocols approved by the Committee of Experimental Animal Administration of Capital Medical University, China (No. AEEI‐2021‐228). Wild‐type and C3 transgenic mice used in all experiments were age‐matched.

### 2.2 Behavioral Tests

#### 2.2.1 Open Field Test

The open field test was conducted in a clear square arena (50 cm × 50 cm × 40 cm; Xinruan Information Technology Co., Shanghai, China) with a camera positioned 150 cm above the box to record mouse behavior. Mice were acclimated in the laboratory for at least 1 h before the experiment to minimize stress and allow adaptation to the environment. During the test, mice were allowed to explore the arena freely for 15 min; their exploratory behavior was recorded and analyzed using Tracking Master 4.4.2 software.

#### 2.2.2 Novel Object Recognition

The novel object recognition test was conducted in a square experimental box (50 cm × 50 cm × 40 cm; Xinruan Information Technology Co.) with a camera placed 150 cm above the box to record mouse behavior. The experiment lasted 5 days. During the first 3 days, mice were given a 15‐min acclimation period each day to explore the box and adapt to the environment. On Day 4, two identical cubes (yellow, 5 × 5 × 4 cm) were placed in the box. On Day 5, one of the cubes was replaced with a cylinder (red, 2.8 × 5.8 cm). The time spent by mice exploring each object during a 5‐min period was recorded and analyzed using Tracking Master 4.4.2.

#### 2.2.3 Fear Conditioning

Fear conditioning was used to assess memory by measuring the tendency of mice to exhibit a fear response (freezing) when re‐exposed to a context where they previously received an aversive stimulus (foot shock), as previously described (Shi et al. 2015). The conditioning chamber (26 cm × 21 cm × 10 cm; Xinruan Information Technology Co.) was constructed with perspex sides, stainless steel end walls, and a steel rod floor. Mice were allowed to explore the chamber for 2 min, with a 2‐min interval between each 2‐s foot shock (0.5 mA). One minute after the final shock, mice were removed from the chamber. Twenty‐four hours after the training session, mice were returned to the conditioning chamber for 5 min, without administering an electric shock. Freezing behavior was recorded using Super Maze software. Chamber floor bars and waste trays were cleaned with an ethanol solution between sessions.

#### 2.2.4 Rotarod Test

The rotarod test is a standard method for assessing motor function in rodents. The experimental setup includes a rotating rod, a stand, a timer, and other instruments. Mice were placed on the rotating rod, which was uniformly accelerated from rest to a speed of 5 rpm; this speed was maintained for 1 min. After the mice had adapted, the speed was increased to 40 rpm within 5 min. The same procedure was repeated four times; the time each mouse remained on the rotating rod was recorded and analyzed.

#### 2.2.5 Y‐Maze

The Y‐maze spontaneous alternation behavior test is used to assess short‐term working memory in mice. During the test, mice were allowed to freely explore the maze for 8 min. Sequentially entering three different arms was considered a successful alternation. Working memory ability was evaluated based on the spontaneous alternation rate, defined as the ratio of successful alternations to the total number of alternations.

The novel arm maze test is a standard method for assessing spatial cognition in mice. This experiment consisted of a training phase and a testing phase. In the training phase, the novel arm was blocked with a partition; mice were placed in an open arm, allowing them to explore freely for 10 min. After a 1‐h interval, the testing phase began. The partition was removed and mice were placed in the same starting position as in the training phase. They were allowed to freely explore the maze for 5 min while time spent in the novel arm was recorded.

#### 2.2.6 Barnes Maze

The Barnes maze test was conducted on a gray circular platform 140 cm high (medical‐grade acrylonitrile butadiene styrene board; inner diameter, 91 cm; containing twenty 5‐cm‐diameter holes). The experiment lasted 5 days. During the first 4 days (training), a hole was randomly designated as the target hole and connected to a target box. This target hole allowed mice to freely explore the maze for 4 min each day; training sessions occurred twice daily. On the fifth day, the target box was removed. The latency for each mouse to locate the target hole, along with the number of incorrect holes explored before reaching the target, was recorded and analyzed.

### 2.3 Enzyme‐Linked Immunosorbent Assay (ELISA)

A Complement C3 Mouse ELISA Kit (ab157711, Abcam, UK) was used for the quantitative determination of complement C3 in mouse plasma, in accordance with the Complement C3 Mouse ELISA Kit's manufacturer's instructions. Blood was collected via the orbital venous plexus into an anticoagulant‐containing container and then centrifuged at 4°C at 1000×*g* for 5 min, and the supernatant was collected for analysis. Plasma was diluted at a 1:50,000 ratio to prepare the test sample. For each sample and standard, 100 μL were added to a 96‐well plate and incubated at room temperature for 20 min.

A mouse C3a ELISA kit (E‐EL‐M0337, Elabscience, China) was used to quantify C3a content in brain tissue. After mouse brain tissue had been weighed, the tissue was homogenized to prepare a 10% homogenate. The homogenate was centrifuged at 4°C at 5000×*g* for 10 min to collect the supernatant. Subsequently, 100 μL of each sample and standard was added to a 96‐well plate and incubated at 37°C for 60 min.

After incubation, the plate was washed four times with wash buffer. Next, 100 μL of 1× enzyme‐antibody conjugate was added to each well and incubated at room temperature in the dark for 20 min. After this incubation, the plate was washed again four times. Then, 100 μL of tetramethylbenzidine substrate solution was added to each well and incubated at room temperature in the dark for 10 min. The reaction was stopped by adding 100 μL of stop solution to each well, and the absorbance at 450 nm was measured with multi‐detection microplate readers (Spectra Max M2, Molecular Devices, China).

### 2.4 Immunofluorescence Staining

Serial 10‐μm‐thick coronal paraffin sections of mouse brain were mounted on glass slides, then deparaffinized with xylene and an ethanol gradient. Brain sections were subjected to antigen retrieval by microwaving in an appropriate solution, followed by washing with phosphate‐buffered saline (PBS). Sections were blocked for 1 h in PBS containing 10% goat serum and 0.3% Triton X‐100, then incubated overnight at 4°C with primary antibodies. Primary antibodies were diluted in PBS as follows: C3 (1:1000, ab200999, Abcam), postsynaptic density protein 95 (PSD95; 1:500, ab18258, Abcam), neuronal nuclear antigen (NeuN; 1:200, ab177487, Abcam), glial fibrillary acidic protein (GFAP; 1:500, ab7260, Abcam), and ionized calcium‐binding adaptor molecule 1 (IBA1; 1:500, ab178847, Abcam). Secondary Alexa Fluor‐conjugated antibodies (1:400, A32790, Thermo Fisher Scientific, USA, and 1:500, ab150080, Abcam) were applied for 1 h at room temperature. 4′,6‐Diamidino‐2‐phenylindole (DAPI; 1:4000, C1002, Beyotime, China) was then used to stain the sections for 10 min before sealing. For statistical analysis, stained brain sections were scanned using a digital pathology system (3Dhistech, Hungary).

### 2.5 Western Blotting

Mouse brain tissues were weighed, and lysis buffer from a fresh radio protein extraction kit (PC3730, Solarbio, China) was added. Tissues were ground on ice until no visible tissue masses remained. Homogenates were centrifuged at 4°C at 12,000×*g* for 30 min, and the supernatant was collected for analysis. Proteins were separated using MOPS‐SDS running buffer (F00001Gel, ACE, China) and transferred to polyvinylidene fluoride membranes (IPVH00010, Millipore Corporation, USA). Membranes were then immersed in a 5% bovine serum albumin solution (in Tris‐buffered saline plus 0.1% Tween‐20, pH 7.2) for 1 h at room temperature, followed by overnight incubation at 4°C with primary antibodies against C3 (1:1000, ab200999, Abcam), C3aR (1:1000, bs‐2955R, Bioss, China), CD11b (1:1000, ab133357, Abcam), insulin receptor (1:2000, ABclonal, China), glyceraldehyde‐3‐phosphate dehydrogenase (GAPDH; 1:5000, MA5‐15738, Thermo Fisher Scientific), and tubulin (1:1000, T0023, Affinibody, China). After extensive washing, membranes were incubated with peroxidase‐labeled goat anti‐mouse or anti‐rabbit IgG (ZB‐2305 and ZB‐2301, ZSGB‐Bio, China). Epitope visualization was performed using an enhanced chemiluminescence Western blot detection kit (P10300, NCM Biotech, China). All procedures followed the manufacturer's instructions for each antibody. Densitometric analysis was conducted using ImageJ software.

### 2.6 Reverse Transcription Quantitative PCR (qPCR)

Primer sequences were designed and sent to a synthesis company (Sangon Biotech, China). Ultra‐low temperature frozen mouse brain samples were weighed and quickly transferred to a pre‐cooled mortar and pestle with liquid nitrogen, then ground into a fine powder. An RNA extraction kit (ER501, TransGen, China) was used to extract RNA from the brain tissue, and a reverse transcription kit (AT301, TransGen) was used to transcribe the extracted RNA into cDNA. Primers, SYBR Green (A57156, Thermo Fisher Scientific), and other reagents were added in accordance with the kit instructions, thoroughly mixed, and aliquoted into a 96‐well qPCR plate along with the extracted cDNA samples, maintaining the appropriate concentration and volume for the reaction system. The 96‐well plate was placed into a thermocycler for PCR cycling using a preset program (1 cycle at 95°C for 2 min followed by 40 cycles at 95°C for 15 s and 60°C for 60 s with QuantStudio 1Plus), which allowed detection of DNA denaturation, primer binding, amplification, and fluorescence signal. Fluorescence signal changes were used to analyze Ct values and calculate relative amounts of target sequences. In the qPCR microarray (WG‐MRNAYTH2310191‐M, WG‐mRNAYTH2310192‐M, WcGene Biotech, China), primers were pre‐added, and the remaining steps followed the qPCR protocol.

### 2.7 Cell Counting Kit‐8 (CCK‐8) Assay

CCK‐8 reagent (C6005, NCM Biotech, China) is used to measure the viability of live astrocyte cell line U251. Astrocytes line were seeded into 96‐well plates at a density of 5000 cells per well. The plates were incubated at 37°C with 5% CO_2_ for 24 h. The medium was then replaced with fresh medium containing either 200 nM C3a (HY‐P7862, MCE, USA), a C3aR competitive antagonists (SB290157, HY‐101502A, MCE), or the respective control medium. The plates were returned to the incubator for an additional 48 h. After incubation, 10 μL of CCK‐8 solution were added to each well. Wells containing only the cell culture medium and CCK‐8 solution, without cells, served as blank controls. Absorbance at 450 nm was measured with multi‐detection microplate readers (Spectra Max M2, Molecular Devices) after 1 h of incubation at 37°C.

### 2.8 Targeted Metabolomics (Absolute Quantitative Analysis of Targeted Metabolic Neurotransmitters in Mouse Brain Tissue)

Brain tissues were collected from six wild‐type and six C3 transgenic mice. From each animal, 50 mg of brain tissue was meticulously excised. Samples were placed in 200 μL of precooled 80% methanol at 4°C and homogenized with a tissue disruptor under low‐temperature conditions. After homogenization, 800 μL of methanol solution at the same temperature was added, and samples were thoroughly mixed with a vortex mixer. The mixtures were ultrasonicated for 20 min in an ice bath to enhance solvent penetration and tissue disruption. After ultrasonication, samples were stored at −20°C for 2 h to facilitate protein precipitation and metabolite release. Samples were then centrifuged at 16,000×*g* for 20 min at 4°C to separate the supernatant from the precipitate. The supernatant was collected and dried using a high‐speed vacuum concentrator to remove solvent and concentrate metabolites. Dried samples were reconstituted in 80 μL of precooled 50% methanol solution and centrifuged at 20,000×*g* for 15 min at 4°C to remove any fine precipitates. The final supernatant was used for mass spectrometry analysis. Mass spectrometry analysis was performed using a QTRAP 5500 mass spectrometer (AB SCIEX) in both positive and negative ion modes. Data were processed with MultiQuant software to identify and quantify metabolites, allowing exploration of neurotransmitter changes.

### 2.9 In Vivo Complement Blockade Therapy

After anesthesia, each mouse was secured in a stereotaxic apparatus, and a cannula was implanted 1 mm above the injection site (anteroposterior: −2.0 mm; mediolateral: −1.5 mm; dorsoventral: −1.5 mm). The cannula was fixed to the skull with two metal screws and dental cement. After surgery, mice were allowed to recover for 3 days, then anesthetized with isoflurane. A syringe pump was used to aspirate and inject 0.2 μg/μL of C3 antibody or IgG (0.2 μL at 0.5 μL/min) once per week for a total of 4 injections. The SB290157 was administered via orbital vein injection. Post‐injection behavioral tests were conducted to assess learning and memory abilities.

### 2.10 Electrophysiology on Ex Vivo Mouse Brain Slices

Mice were anesthetized and transcardially perfused with cold artificial cerebrospinal fluid (ACSF) and the brain was rapidly removed. ACSF contains the following (in mM): 117 NaCl, 3.6 KCl, 1.2 NaH_2_PO_4_·2H_2_O, 2.5 CaCl·2H_2_O, 1.2 MgCl_2_·6H_2_O, 25 NaHCO_3_, and 11 glucose, pH 7.4. Hippocampal tissues were horizontally sliced into 400 μm sections on a vibratome (DTK‐1000, DOSAKA, JPN). Then, brain slices were incubated in ACSF that had been saturated with 95% O_2_ and 5% CO_2_ for 60 min.

Multi‐electrode array (MED64) (SU‐MED640, Alpha Med Science, JPN) is used to record field EPSPs (fEPSPs) of CA3–CA1 synapses in the hippocampal tissues. Post‐incubation, the MED probe (MED‐P515A, Alpha Med Science, JPN) was positioned in the Schaffer collaterals for stimulation and records. The slice was perfused with the same fresh ACSF at a rate of 0.5–2 mL/min with the aid of a peristaltic pump (BT100‐2J, Longer, CHN) through the whole period of electrophysiological recording. Initially, the brain slice's input/output (I/O) curve was captured to determine its optimal stimulation. During the induction of long‐term potentiation (LTP), brain slices were stimulated with five pulses (const 5 ms, pulse 0.2 ms, const 9.8 ms) and continuously recorded.

### 2.11 Reactive Oxygen Species Fluorometric Assay

Intracellular reactive oxygen species (ROS) levels were measured using a ROS fluorometric assay kit (E‐BC‐K138‐F, Elabscience, China). Astrocytes were seeded in six‐well plates. After removal of the culture medium, cells were incubated with 1 mL 2,7‐dichlorofuorescin diacetate (DCFH‐DA) at 37°C in the dark for 30 min. Subsequently, the DCFH‐DA solution was removed, and cells were washed three times with serum‐free medium to eliminate any residual probe. Cells were then treated with trypsin, and the resulting suspension was centrifuged at 1000×*g* for 5 min to collect the cells. Finally, the cell pellet was resuspended in serum‐free medium for fluorescence measurement.

### 2.12 Statistical Analysis

Bar charts of behavioral test results and immunofluorescence staining for the two animal groups were analyzed with Student's *t*‐test in GraphPad Prism 9.0 (GraphPad Software, San Diego, California, USA). For animal behavior experiments, two‐way analysis of variance (ANOVA) was used to display line graphs of *p*‐values over time. For comparisons among three data sets, one‐way ANOVA was utilized. All data were presented as mean ± standard error of the mean.

## 3 Results

### 3.1 Increased C3 Expression in C3 Transgenic Mice

**FIGURE 1:**
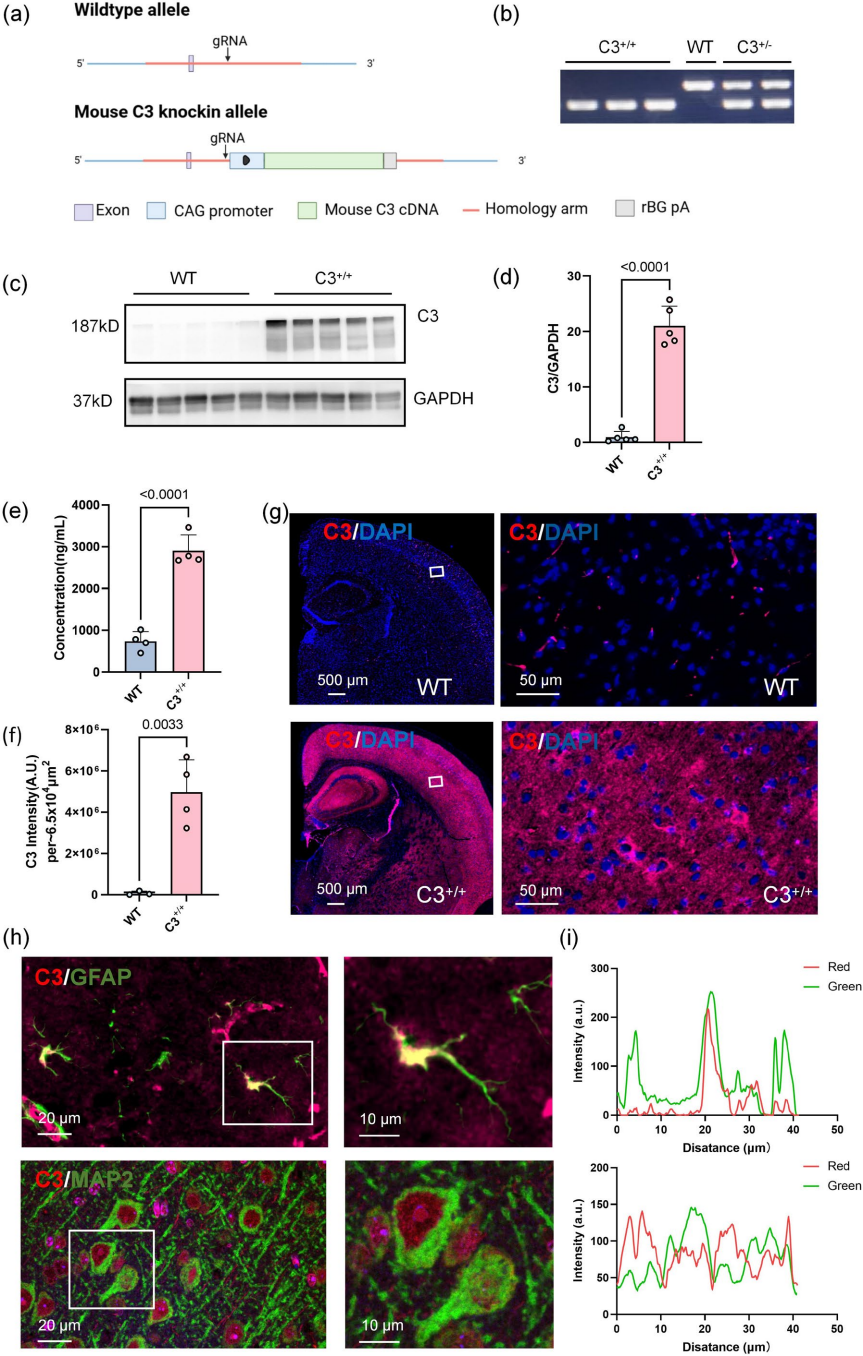
Elevated C3 expression levels in brain tissue and plasma of C3 transgenic mice. (a) Schematic diagram of the construction of genetically engineered C3 transgenic mice. (b) PCR analysis of transgenic mice. (c, d) Western blotting analysis of C3 in 6‐month‐old C3^+/+^ mice (*n* = 5) and WT mice (*n* = 5) (unpaired Student's *t*‐test). (e) ELISA analysis of plasma from C3 transgenic mice (*n* = 4) and WT mice (*n* = 4) (unpaired Student's *t*‐test). (f, g) Representative images of brain immunostaining for complement C3 in 6‐month‐old WT (*n* = 3) and C3 transgenic mice (*n* = 4) (unpaired Student's *t*‐test). (h) Representative images of co‐staining of C3 (red) with a neuronal marker (MAP2, green) and an astrocyte marker (GFAP, green). (i) Fluorescence intensity analysis of panel h. All data are presented as mean ± standard deviation (SD).

To investigate the role of elevated C3 in learning and memory impairments, we successfully generated C3 transgenic mice using CRISPR‐Cas9 technology. Figure 1a illustrates the construction of C3 transgenic mice. We screened for C3 transgenic mice using PCR, identifying heterozygous mice by two bands; the lower band indicated homozygosity, and the upper band indicated wild‐type (Figure 1b). After identifying C3 transgenic mice, we assessed changes in protein levels. Western blotting analysis showed a significant increase in C3 levels in the brain tissue of homozygous mice (*p* < 0.0001) (Figure 1c,d). Additionally, we measured C3 protein levels in the plasma and brain of wild‐type and C3 transgenic mice using ELISA and immunofluorescence analysis. ELISA revealed a significant increase in C3 expression in the plasma of C3 transgenic mice (*p* < 0.0001) (Figure 1e). A similar result was observed in brain tissue, where immunofluorescence indicated a significant increase in C3 expression in the brains of C3 transgenic mice relative to the control group (i.e., wild‐type mice) (*p* < 0.0033) (Figure 1f,g). To further examine C3 expression in specific cell types, we used immunofluorescence co‐staining with GFAP, an astrocyte activation marker, and MAP2, a neuronal marker, in addition to C3. As shown in Figure 1h, red fluorescence represents C3, whereas green fluorescence denotes GFAP or MAP2. By analyzing the fluorescence intensity in 40 μm regions, we confirmed that complement C3 is expressed in astrocytes and neurons (Figure 1i). These findings reveal a significant increase in C3 protein expression in both brain tissue and plasma of C3 transgenic mice, mimicking the age‐related increase in complement C3 observed among older people. These results provide a foundation for further investigation into the impact of elevated C3 expression on learning and memory.

### 3.2 Learning and Memory Impairments in C3 Transgenic Mice

**FIGURE 2:**
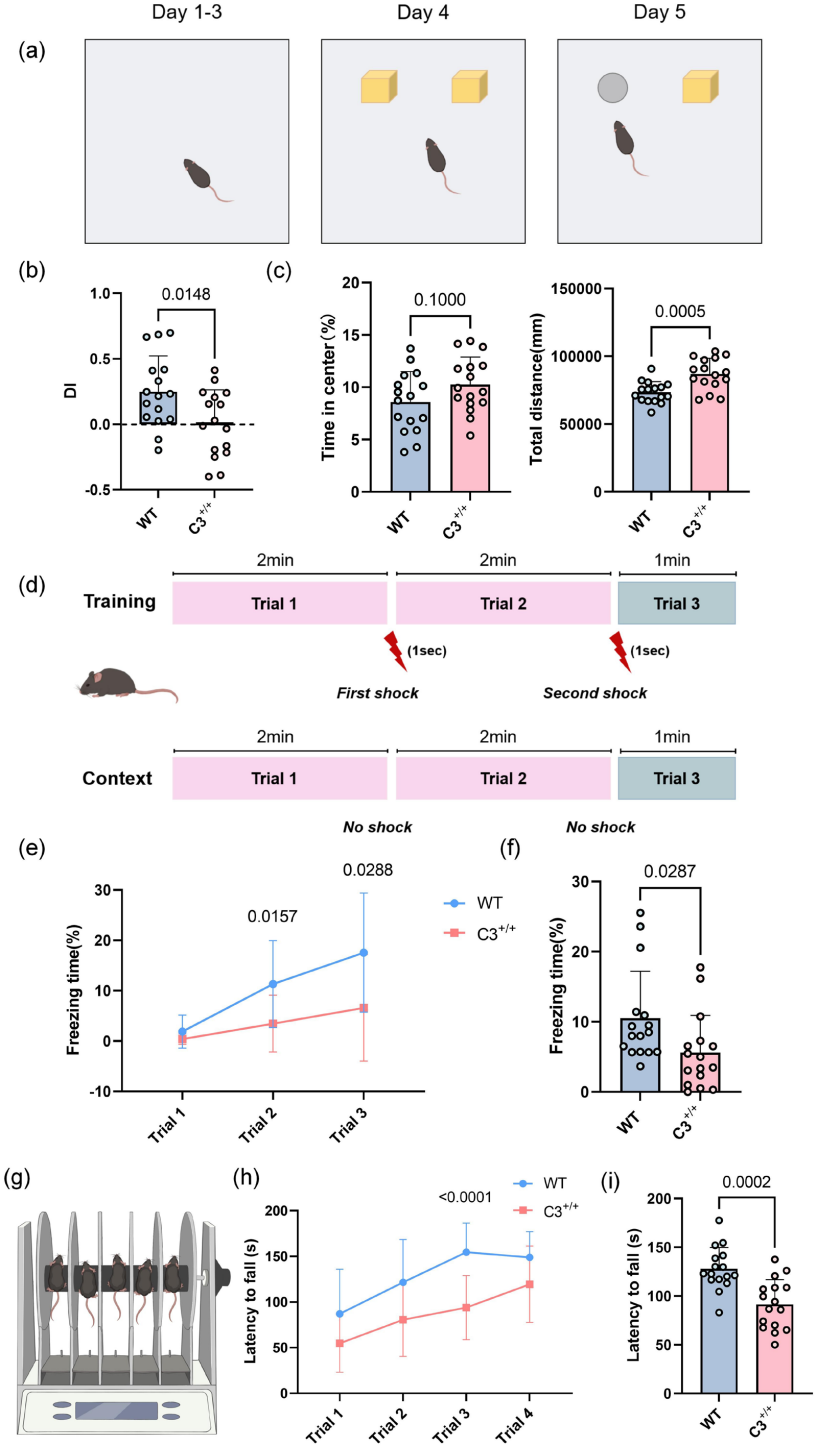
Impaired learning and memory in 6‐month‐old C3 transgenic mice. (a) Schematic illustration of the novel object recognition test. (b) Discrimination index in the novel object recognition test for 6‐month‐old C3 transgenic and age‐matched wild‐type mice (mean data from *n* = 16 [WT] and *n* = 16 [C3^+/+^] animals per group; unpaired Student's *t*‐test). DI = (time spent exploring the novel object—time spent exploring the familiar object)/(time spent exploring the novel object + time spent exploring the familiar object). (c) Time spent in the center and total distance traveled in the open field test for 6‐month‐old WT and C3 transgenic mice (*n* = 16 [WT] and *n* = 16 [C3^+/+^] animals per group; unpaired Student's *t*‐test). (d) Schematic illustration of the fear conditioning test. (e, f) Quantification of the percentage of freezing behavior in animals after contextual fear conditioning (*n* = 16 [WT] and *n* = 16 [C3^+/+^] animals per group; two‐way ANOVA for training and unpaired Student's *t*‐test for context). (g–i) Mean fall latency time in the rotarod test for C3 transgenic and WT mice (*n* = 16 [WT] and *n* = 16 [C3^+/+^] animals per group; two‐way ANOVA for panel h and unpaired Student's *t*‐test for panel i. All data are presented as mean ± SD.

To examine the effects of elevated C3 expression on learning and memory, we conducted behavioral tests on 16 wild‐type mice and 16 C3 transgenic mice. The novel object recognition test assesses cognitive memory by measuring the time spent exploring familiar and novel objects. Figure 2a shows the process of the novel object recognition experiment, which included a 3‐day habituation period. On the fifth day, one of the familiar objects was replaced with a novel object, and memory was assessed using the discrimination index (DI). The results indicated that the DI of C3 transgenic mice was significantly lower than that of wild‐type mice (*p* = 0.0148), suggesting that elevated C3 expression impairs memory (Figure 2b). Additionally, we evaluated anxiety‐like behavior and activity levels through open field testing. The anxiety levels of C3 transgenic mice did not significantly differ from those of the control group (*p* = 0.1000). However, C3 transgenic mice displayed significantly higher activity levels in the open field compared with the control group (*p* = 0.0005) (Figure 2c).

The fear conditioning test was used to assess learning and memory abilities throuhgh the rigid behavioral responses of mice to fear‐inducing stimuli, divided into two stages: training and context (Figure 2d). Compared with the control group, C3 transgenic mice showed significant reductions in freezing duration during trials 2 and 3 (*p* = 0.0157 and *p* = 0.0288, respectively) (Figure 2e). In the context phase, C3 transgenic mice also had a significantly shorter freezing time than wild‐type mice (*p* = 0.0287) (Figure 2f). These results suggest that C3 transgenic mice have significantly impaired fear learning ability. Aging is often associated with a decline in motor abilities; thus, we used the rotarod test to assess limb coordination in mice (Figure 2g). The rotarod results showed that the mean fall latency during the third trial was significantly shorter among C3 transgenic mice than among control mice (*p* < 0.0001) (Figure 2h). Additionally, the overall mean fall latency across four trials was shorter in C3 transgenic mice than in control mice (*p* = 0.0002) (Figure 2i). In order to explore the potential effects of elevated C3 expression on brain development, we found that 3‐month‐old C3 transgenic mice exhibited comparable cognitive behavior to wild‐type mice, and immunofluorescence revealed no pathological changes in the brains of 1‐month‐old transgenic mice (Figure S1). In summary, these results demonstrate that elevated C3 expression impairs learning, memory, and motor abilities in mice.

### 3.3 Neuronal Loss, Synaptic Loss, and Astrocytosis in C3 Transgenic Mice

**FIGURE 3:**
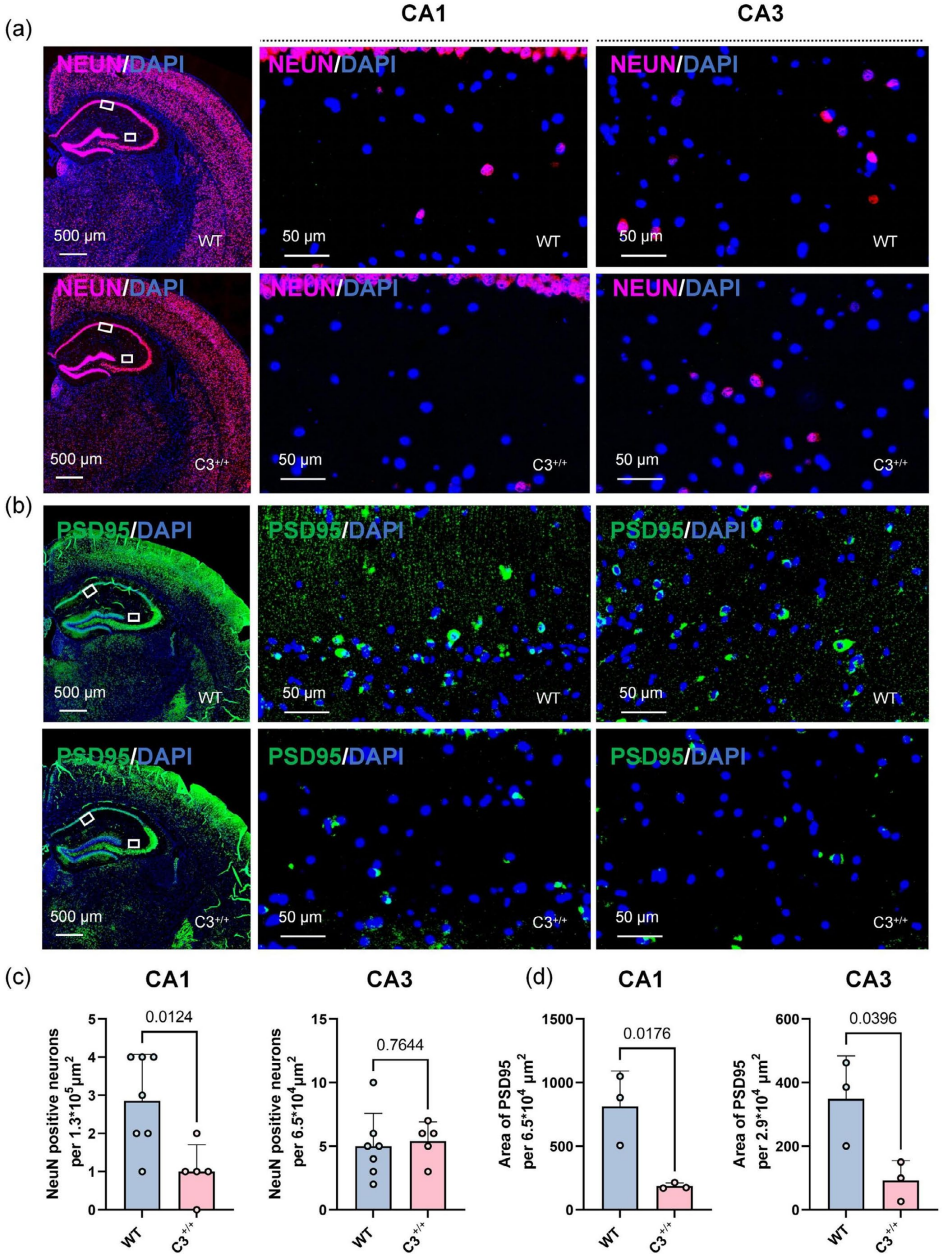
Synaptic and neuronal loss in C3 transgenic mice. (a, b) Representative immunofluorescence images of hippocampal sections from 7‐ to 8‐month‐old C3 transgenic mice and age‐matched wild‐type (WT) mice, showing immunostaining with NeuN (pink) for neurons and PSD95 (green) for synapses. (c) Quantification of NeuN‐positive cells in the hippocampus of WT (*n* = 7) and C3 transgenic mice (*n* = 5) (unpaired Student's *t*‐test). (d) Quantification of PSD95 fluorescence area in the hippocampus of WT (*n* = 3) and C3 transgenic mice (*n* = 3) (unpaired Student's *t*‐test). Scale bars represent 500 and 50 μm. All data are presented as mean ± SD. Individual data points represent biological replicates.

There are potential correlations between deficits in learning and memory and changes in the number of hippocampal synapses and neurons (Yang et al. 2019). To better understand how elevated C3 expression affects neurons and synapses, we used immunofluorescence to detect levels of the neuronal marker NeuN and the postsynaptic density protein marker PSD95 in the mouse hippocampus. Immunofluorescence results showed that elevated C3 expression led to a significant decrease in the number of neurons in the CA1 region (*p* = 0.0124), whereas the number of neurons in the CA3 region of the hippocampus did not show a significant change (*p* = 0.7644) (Figure 3a,c). Additionally, PSD95 staining revealed significant reductions in PSD95 area within the CA1 and CA3 regions among C3 transgenic mice relative to wild‐type mice (*p* = 0.0176 and *p* = 0.0396, respectively) (Figure 3b,d). These findings indicate that increased C3 expression leads to the loss of neurons and synapses.

**FIGURE 4:**
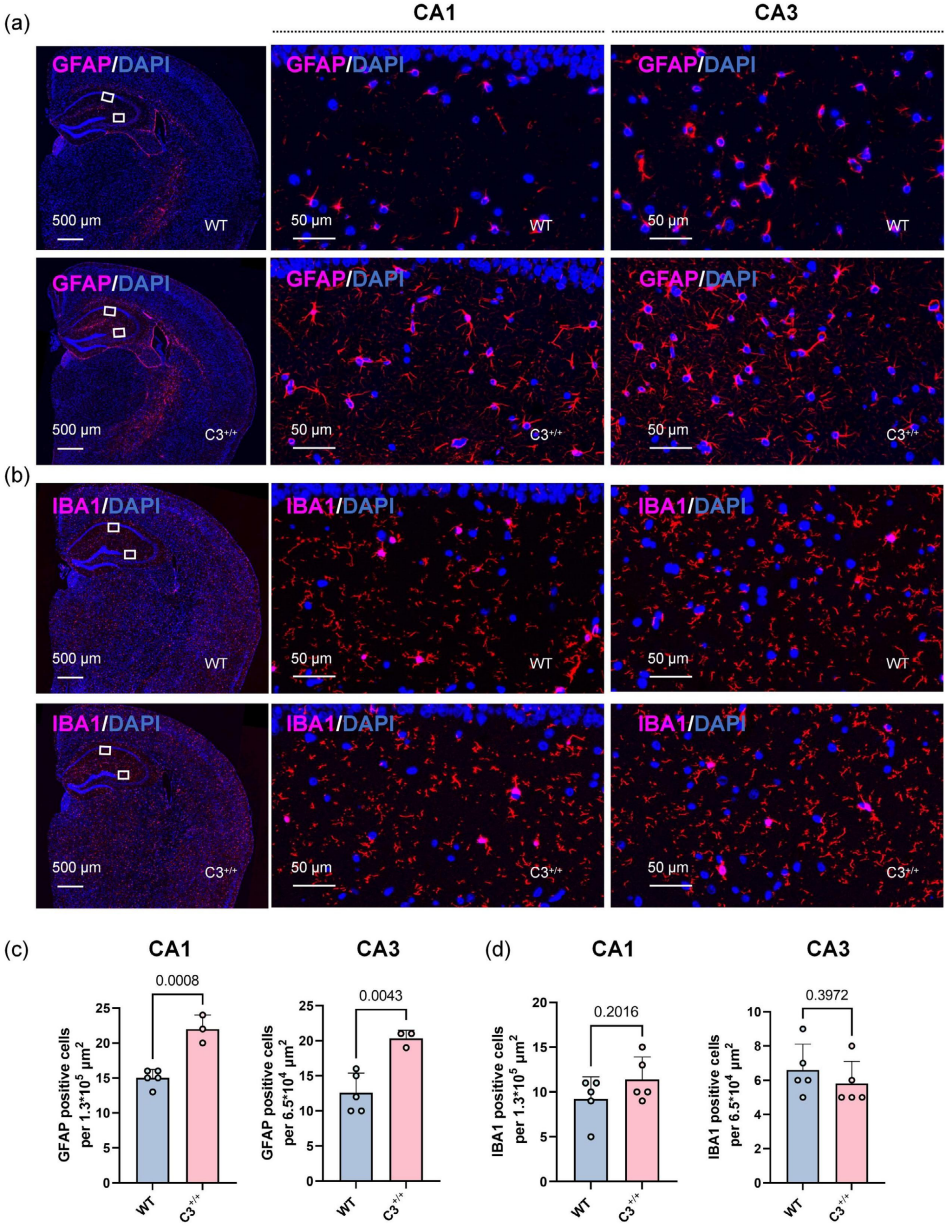
Astrocytosis in C3 transgenic mice. (a, b) Representative immunofluorescence images of hippocampal sections from 7‐ to 8‐month‐old C3 transgenic mice and age‐matched WT mice, showing immunostaining with GFAP (pink) for astrocytes and IBA1 (pink) for microglia. (c) Quantification of GFAP‐positive cells in the hippocampus of WT mice (*n* = 5) and C3 transgenic mice (*n* = 3) (unpaired Student's *t*‐test). (d) Quantification of IBA1‐positive cells in the hippocampus of WT mice (*n* = 5) and C3 transgenic mice (*n* = 5). Scale bars represent 500 and 50 μm. All data are presented as mean ± SD. Individual data points represent biological replicates.

Neuronal and synaptic losses are often accompanied by neuroinflammation. Accordingly, we examined glial cell proliferation in C3 transgenic mice. GFAP serves as a marker for astrocytes, whereas IBA1 is commonly used as a marker for microglia. Immunofluorescence analysis showed a significant increase in the number of GFAP‐positive astrocytes within the CA1 and CA3 regions among C3 transgenic mice (*p* = 0.0008 and *p* = 0.0043, respectively) (Figure 4a,c). No pronounced differences in the number of IBA1‐positive microglia in the CA1 and CA3 regions were observed between the two groups (*p* = 0.2016 and *p* = 0.3972, respectively) (Figure 4b,d). In conclusion, increased C3 expression leads to neuronal and synaptic losses, as well as glial cell proliferation, in mice.

### 3.4 Differential Molecular Characteristics and Impaired Insulin Receptor Signaling in Astrocytes

To further characterize the impact of elevated C3 expression on mRNA levels in astrocytes and microglia, qPCR microarrays were used to measure mRNA levels in these glial cells. Figure S2a shows the 90 mRNAs detected in astrocytes. Compared with wild‐type mice, eight mRNA levels were significantly downregulated and two were upregulated in C3 transgenic mice (fold change < 1 for downregulated and > 1 for upregulated) (Figure S2c). Because differential mRNAs in the qPCR microarray were initially screened using a single sample, further validation was necessary. Validation results indicated that, among six mRNAs tested by qPCR, three showed significant changes (Figure S2e). For microglial cells, molecular detection using a qPCR microarray is shown in Figure S2b. In this microarray, seven differential mRNAs were identified; three were downregulated, whereas four were upregulated in C3 transgenic mice (fold change < 1 for downregulated and > 1 for upregulated) (Figure S2d). For mRNAs exhibiting variation in a single sample, additional samples were tested to confirm the findings. qPCR results showed that, among the four validated differential mRNAs, only one exhibited a consistent change (Figure S2f). In summary, elevated C3 expression alters specific mRNA levels in astrocytes and microglia within the brains of C3 transgenic mice. Insulin receptor substrate 2 (IRS2) is a cytoplasmic signaling molecule that mediates the effects of insulin, insulin‐like growth factor 1, and other cytokines by bridging various receptor tyrosine kinases with downstream effectors (Manohar et al. 2020). In our C3 transgenic mice, IRS2 mRNA levels were altered, suggesting that C3 contributes to learning and memory impairments through its effects on glucose metabolism.

**FIGURE 5:**
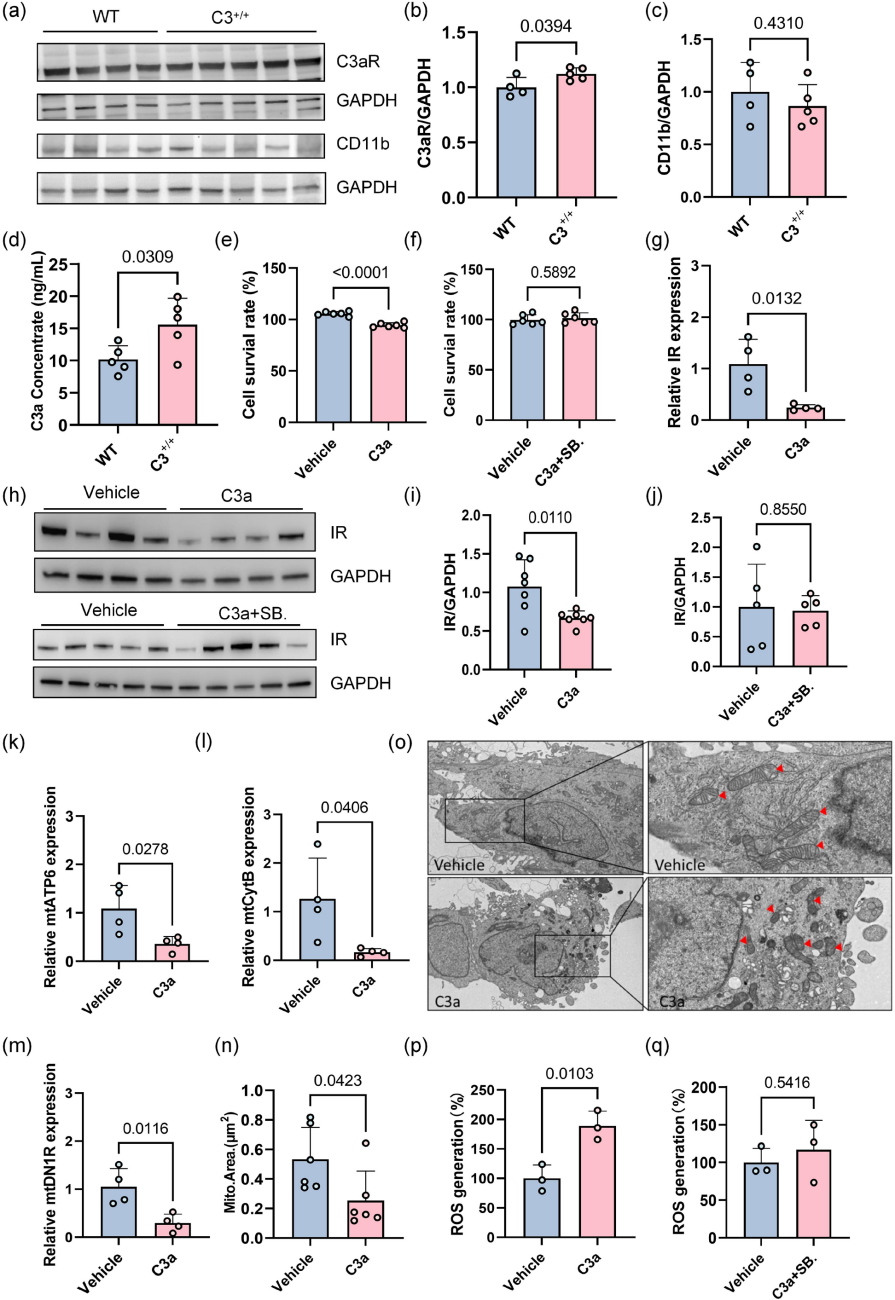
Impairment of astrocyte insulin signaling and mitochondrial structure after activation of the C3a‐C3aR pathway. (a–c) Western blotting analysis of C3aR and CD11b expression in wild‐type (WT, 6 months old) and C3^+/+^ mice (6 months old) (*n* = 4 WT, *n* = 5 C3^+/+^; unpaired Student's *t*‐test). (d) ELISA analysis of C3a levels in brain tissue from WT (*n* = 5) and C3 transgenic mice (*n* = 5) (unpaired Student's *t*‐test). (e, f) CCK‐8 assay of U251 cell survival after C3a (200 nM) and C3a + SB290157 treatments (*n* = 6 per group; unpaired Student's *t*‐test). (g) qPCR analysis of insulin receptor (IR) mRNA levels in U251 cells after C3a treatment (*n* = 4 per group; unpaired Student's *t*‐test). (h–j) Western blotting analysis of IR protein expression in U251 cells after C3a (200 nM) (*n* = 7 per group ) and C3a + SB290157 treatments (*n* = 5 per group; unpaired Student's *t*‐test). (k–m) qPCR analysis of mitochondrial function‐related genes in U251 cells (*n* = 4 per group; unpaired Student's *t*‐test). (n) Quantitative analysis of mitochondrial area (unpaired Student's *t*‐test). (o) Schematic representation of mitochondrial morphology as observed by transmission electron microscopy. (p, q) Measurement of reactive oxygen species (ROS) production in U251 cells: (p) C3a versus vehicle control; (q) C3a + C3aR antagonist (SB290157, 100 nM) versus vehicle control (unpaired Student's *t*‐test). All data are presented as mean ± standard deviation (SD).

To more fully understand how increased C3 expression affects insulin receptor signaling, we examined downstream components of the complement pathway. Considering that C3 can be cleaved into C3a and C3b, it may exert downstream functions through either C3a or C3b. We used Western blotting to measure the levels of C3a receptor (C3aR) and C3b receptor (CD11b) in the brain tissue of C3 transgenic mice. The results showed a significant increase in C3aR expression within C3 transgenic mice relative to wild‐type mice (*p* = 0.0394), whereas CD11b expression levels did not significantly differ between the two groups (*p* = 0.4310) (Figure 5a–c). ELISA analysis confirmed that C3a levels were significantly higher in brain tissues of C3 transgenic mice relative to wild‐type mice (*p* = 0.0309) (Figure 5d). Thus, increased C3 expression may impact astrocytes primarily through elevated C3a. Accordingly, we added C3a to the astrocyte cell line U251 and measured its survival rate. CCK‐8 data indicated that cell viability with added C3a was significantly lower than that of the solvent control group. When both C3a and C3aR competitive antagonist were added concurrently, U251 cell viability did not significantly differ from the vehicle group, suggesting that increased C3 expression indeed affects astrocytes (*p* < 0.0001 and *p* = 0.5892, respectively) (Figure 5e,f).

Insulin receptors are widely distributed in the brain, where insulin regulates various neural activities, including cognition and emotion. Individuals with diabetes and obesity show higher rates of depression, anxiety, cognitive decline, and dementia, many of which are linked to impaired insulin signaling in the brain (Kleinridders et al. 2014; Liu et al. 2011; Talbot et al. 2012). Recent studies indicate that active insulin receptors are expressed in astrocytes; the absence of insulin receptors in astrocytes reduces their adenosine triphosphate (ATP) release capability, exacerbating AD‐like symptoms in 5XFAD mice (Cai et al. 2018; Chen et al. 2023). We used qPCR to measure insulin receptor mRNA levels in U251 cells within 48 h of C3a treatment. The results showed that insulin receptor mRNA levels in U251 cells significantly decreased after C3a treatment relative to vehicle treatment (*p* = 0.0132) (Figure 5g). Furthermore, Western blotting analysis of insulin receptor protein expression in C3a‐treated U251 astrocytic cells yielded consistent results, with significantly lower insulin receptor expression relative to vehicle‐treated controls (*p* = 0.0110) (Figure 5h,i). In contrast, co‐treatment of U251 cells with C3a and the C3aR antagonist SB290157 resulted in insulin receptor protein levels that were not significantly different from those of vehicle‐treated controls (*p* = 0.8550) (Figure 5j). Mitochondrial function within astrocytes is widely regarded as crucial for brain health, and mitochondrial dysfunction is often observed in neurodegenerative diseases (Bell et al. 2021). Studies have revealed that in mature astrocytes, the absence of insulin receptors significantly reduces the expression of genes encoded by mitochondrial DNA (mtDNA) (Cai et al. 2018; Chen et al. 2023). Using qPCR, we assessed the expression of mtDNA‐encoded genes in U251 cells treated with C3a. Our results indicated that the mRNA levels of mtATP6, mtCytB, and mtDN1R were significantly lower in C3a‐treated cells than in the solvent control group (*p* = 0.0278, *p* = 0.0406, and *p* = 0.0116, respectively) (Figure 5k–m).

To further evaluate the impact of C3a on mitochondrial ultrastructure in U251 cells, we utilized transmission electron microscopy (TEM) to examine mitochondrial morphology. TEM analysis revealed significant morphological alterations in C3a‐treated U251 cells compared with vehicle controls, including irregular membrane structures and substantial reduction in mitochondrial area (*p* = 0.0423) (Figure 5n,o). Mitochondrial cristae, essential components of the inner membrane, play a critical role in energy metabolism, particularly in the efficiency of oxidative phosphorylation (Huang et al. 2023). Mitochondrial morphological abnormalities are frequently linked to metabolic dysregulation, elevated ROS production, and apoptotic pathway activation (Guan et al. 2025). Subsequently, we measured the levels of ROS in C3a‐treated U251 cells. C3a treatment significantly increased ROS generation compared with vehicle controls (*p* = 0.013) (Figure 5p). To determine whether this effect was mediated by C3aR, we co‐administered the competitive C3aR antagonist SB290157. This antagonist completely abolished the C3a‐induced increase in ROS, indicating that C3a‐C3aR signaling directly modulates both mitochondrial morphology and ROS production (*p* = 0.5416) (Figure 5q).

The absence of insulin receptors in astrocytes has been shown to modulate the activity of midbrain dopaminergic neurons (Chen et al. 2023). To determine whether increased C3 expression affects neurotransmitter release by neurons, we randomly selected 12 mice (six wild‐type and six C3 transgenic) for targeted neurotransmitter metabolism analysis. Figure S3a depicts the hierarchical clustering results of differentially expressed metabolites between the groups. Cluster analysis revealed significant within‐group variation in neurotransmitter expression (Figure S3a). Orthogonal partial least squares discriminant analysis (OPLS‐DA), a supervised technique for discriminant analysis, indicated substantial differences in neurotransmitter metabolism between the two groups (Figure S3b,c). As shown in Figure S2d, dopamine levels in the brains of C3 transgenic mice were significantly decreased, consistent with previous findings that a loss of insulin signaling in astrocytes leads to dopamine depletion (Chen et al. 2023). These results suggest that elevated complement C3 expression in the brain increases C3a levels, resulting in a reduction of insulin receptors among astrocytes. This reduction affects mitochondrial function in astrocytes and dopamine release, ultimately impacting learning and memory.

### 3.5 Targeting Brain Complement C3 Improves Learning and Memory in SAMP8 Mice

**FIGURE 6:**
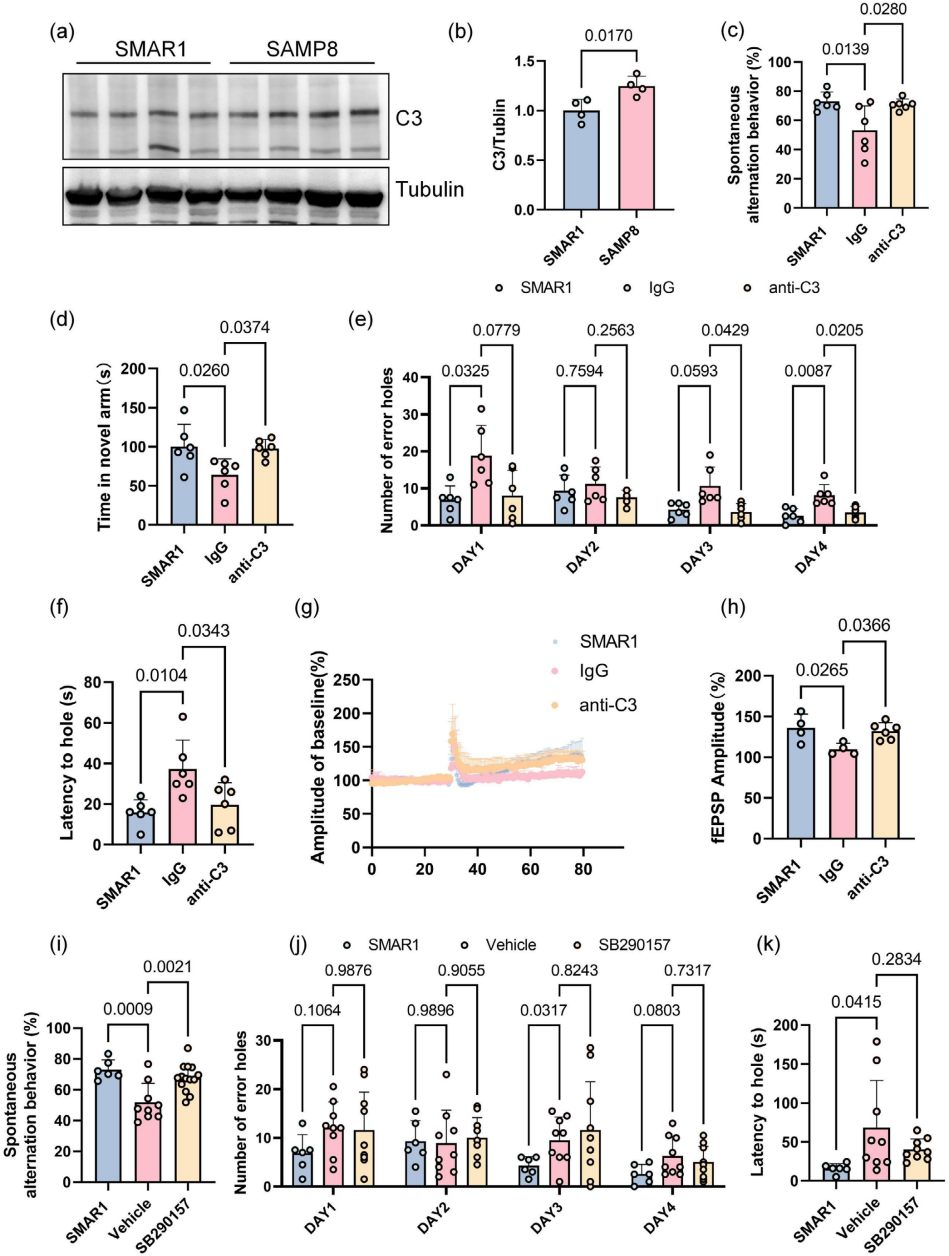
Improvements in learning and memory among SAMP8 mice after targeting brain complement C3. (a, b) Western blotting analysis of C3 expression in SMAR1 and SAMP8 mice (*n* = 4 per group; one‐way ANOVA). (c) Spontaneous alternation behavior in the Y‐maze following antibody blockade in 5‐month‐old SMAR1 mice (*n* = 6), IgG‐treated mice (*n* = 6), and anti‐C3‐treated mice (*n* = 6) (one‐way ANOVA). (d) Time spent in the novel arm during the Y‐maze test in 5‐month‐old SMAR1 mice (*n* = 6), IgG‐treated mice (*n* = 6), and anti‐C3‐treated mice (*n* = 6) (one‐way ANOVA). (e, f) Number of errors and latency to find the target hole in the Barnes maze test for 5‐month‐old SMAR1 mice (*n* = 6), IgG‐treated mice (*n* = 6), and anti‐C3‐treated mice (*n* = 6) (one‐way ANOVA). (g, h) fEPSP amplitude of LTP in 5‐month‐old SMAR1 mice (*n* = 4 slices), IgG‐treated mice (*n* = 4 slices), and anti‐C3‐treated mice (*n* = 6 slices) (one‐way ANOVA) (i) Spontaneous alternation behavior in the Y‐maze after orbital vein injection of antagonist in 5‐month‐old SMAR1 mice (*n* = 6), vehicle‐treated mice (*n* = 9), and SB290157‐treated mice (*n* = 14) (one‐way ANOVA). (j, k) Number of errors and latency to find the target hole in the Barnes maze test for 5‐month‐old SMAR1 mice (*n* = 6), vehicle‐treated mice (*n* = 9), and SB290157‐treated mice (*n* = 9) (one‐way ANOVA). All data are presented as mean ± SD.

To investigate the effect of antibody‐mediated complement C3 blockade on learning and memory improvement in mice, we first identified an aged mouse model with increased complement C3 expression. The SAMP8 mouse model was selected due to its widespread use in studies of AD and other aging‐related neurodegenerative diseases, as well as its recognized deficits in learning and memory abilities. The SAMR1 mouse serves as a normal‐aging control for the SAMP8 strain. These two strains share an identical genetic background. In contrast to rapidly aging SAMP8 mice, SAMR1 mice exhibit a normal aging process (Cheng et al. 2014; Dacomo et al. 2024; Dai et al. 2024). In this study, SAMR1 mice were used as normal‐aging controls. Western blotting analysis revealed significantly higher levels of complement C3 in the brain of 4‐month‐old SAMP8 mice compared with the control group, making it an ideal model for antibody blockade therapy (*p* = 0.0170) (Figure 6a,b). We implanted a cannula in the mice and performed stereotaxic brain surgery to administer the complement C3 antibody (ab200999) into the brain tissue once per week for a total of four injections. After treatment, the working memory of 5‐month‐old mice was assessed using the Y‐maze spontaneous alternation test. The results showed that, after IgG injection, the spontaneous alternation rate was significantly lower in SAMP8 mice than in the SMAR1 group (*p* = 0.0139). Compared with the IgG group, antibody‐treated SAMP8 mice showed a higher spontaneous alternation rate (*p* = 0.0280), indicating that C3 antibody effectively blocked complement C3 in brain tissue, thereby significantly improving the working memory capacity of SAMP8 mice (Figure 6c). Additionally, the Y‐maze novel arm test was used to assess spatial memory. The results of the novel arm test indicated that, compared with the IgG group, both SMAR1 and antibody‐treated SAMP8 mice spent significantly more time exploring the novel arm (*p* = 0.0260 and *p* = 0.0374, respectively) (Figure 6d), suggesting that antibody treatment improved spatial learning and memory in SAMP8 mice. The Barnes maze, a classic paradigm for assessing spatial learning and memory, was also used to test 5‐month‐old SAMP8 mice after antibody treatment. On test day, the latency to find the target hole was significantly lower in both SMAR1 and C3 antibody‐treated SAMP8 mouse groups compared with the IgG group (*p* = 0.0104 and *p* = 0.0343, respectively) (Figure 6f). During the 4‐day training, SMAR1 and C3 antibody‐treated SAMP8 mice showed a significantly lower number of errors before locating the target hole on Day 4, relative to IgG‐treated SAMP8 mice (*p* = 0.0087 and *p* = 0.0205, respectively) (Figure 6e). Field recording of the CA3–CA1 Shaffer collateral LTP elicited by high‐frequency stimulation was performed on brain slices. MED64 results showed a significant reduction of LTP in both SMAR1 and C3 antibody‐treated SAMP8 mouse groups compared with the IgG group (*p* = 0.0265 and *p* = 0.0366, respectively) (Figure 6g,h). This experiment also revealed that the injection of a complement C3aR competitive antagonist via the orbital vein produced similar improvements in Y‐maze working memory. Using SMAR1 as the negative control, we observed a significant increase in spontaneous alternation rate among SAMP8 mice treated with the antagonist relative to the vehicle‐injected group, indicating that the antagonist also positively affects working memory (*p* = 0.0009 and *p* = 0.0021, respectively) (Figure 6i). However, Barnes maze test results for antagonist‐treated mice showed no reduction in the number of incorrect holes explored and no improvement in latency to find the target hole during training (*p* = 0.2834), suggesting that antagonist treatment did not affect spatial learning and memory (Figure 6j,k). In summary, antibody blockade of complement C3 in the brain significantly improves the learning and memory abilities of SAMP8 mice, providing strong evidence that complement C3 could be a potential therapeutic target for enhancing learning and memory.

## 4 Discussion

The immune system plays a crucial role in shaping the brain during development; the nervous and immune systems both undergo substantial changes with age (Amor and Woodroofe 2014). C3, a central component of the complement system, increases in the brain with age and is further elevated in patients with neurodegenerative diseases and corresponding animal models (Schartz and Tenner 2020; Zhou et al. 2008). However, it remains unclear whether increased complement C3 expression directly contributes to learning and memory impairments. In this study, we developed a transgenic mouse model that mimics the age‐related increase in C3 expression. Through a range of behavioral, molecular, and cellular analyses, we confirmed that elevated C3 expression leads to cognitive decline, synaptic and neuronal loss, astrocytosis, insulin signaling impairment in astrocytes, and mitochondrial dysfunction. Notably, by blocking complement C3 in the brains of SAMP8 mice using antibodies, we were able to effectively reverse these learning and memory impairments. These findings suggest that elevated complement C3 levels contribute to age‐related cognitive decline by modulating insulin signaling.

Initially, the liver was considered the primary site for complement protein synthesis (Perlmutter and Colten 1986). However, subsequent research has shown that complement proteins are also produced in various cell types in the brain during development, as well as in different tissues (Gomez‐Arboledas et al. 2021; Perlmutter and Colten 1986; Schartz and Tenner 2020). Resident neurons, astrocytes, microglia, and oligodendrocytes all can express complement components during brain development or in response to injury and aging (Zhou et al. 2020). In adult human and mouse brains, microglia mainly produce C1q, which increases with age. In APP mice, classical pathway C4 co‐localizes with oligodendrocytes, not astrocytes or microglia, while complement component C3 co‐localizes with astrocytes (Fonseca et al. 2017; Zhou et al. 2008). In our study, we observed a significant increase in complement C3 in both the serum and brain tissue of C3 transgenic mice, with substantial upregulation of C3 expression in brain tissue (e.g., astrocytes and neurons). This finding is consistent with reports of C3 co‐localization in brain tissue and astrocytes.

Complement components are implicated in age‐related changes and various neurodegenerative diseases. C3 deficiency reportedly prevents age‐related synaptic and neuronal loss in specific brain regions and protects against cognitive impairment in aging C57BL/6 mice. C3 deficiency also protects against neurodegeneration in aged APP/PS1 mice (Shi et al. 2017). In human AD, complement C3 is significantly upregulated and associated with amyloid plaques. There is evidence that 16‐month‐old APP/PS1 mice lacking C3, despite their increased quantities of Aβ plaques, perform significantly better on learning and memory tasks than APP/PS1 mice with normal C3 levels (Shi et al. 2017). These findings suggest that C3 deficiency enhances learning and memory in mice, but this hypothesis does not require C3 to directly cause cognitive impairments. In our study, transgenic mice with elevated C3 expression showed worse performance on learning and memory tasks, such as novel object recognition and conditioned fear, at 6 months of age relative to the control group. These results do not conflict with previous findings; rather, they suggest that C3 is a key factor contributing to cognitive impairments during aging.

Synaptic degeneration is an early indicator of aging and neurodegenerative diseases, often occurring before neuronal loss. In AD, early synaptic loss is closely linked to cognitive decline. Complement proteins are involved in synaptic pruning during brain development, where they mediate the phagocytosis of excess or inactive synapses to refine synaptic circuits (Schafer et al. 2012). Complement C3b binds to the CR3 receptor on microglia, facilitating microglial phagocytosis. The first 2 weeks after birth represent a critical period for synaptic remodeling. During this phase, these synapses are selectively eliminated, while the remaining synapses undergo refinement and strengthening (Stephan et al. 2012). Despite persistent deficits in synaptic pruning by mice deficient in complement proteins C1q or C3, these still undergo a substantial degree of synapse elimination, indicating that the complement system cooperates with other molecular pathways (Stephan et al. 2012; Stevens et al. 2007). Our study shows that elevated C3 expression does not significantly affect neuronal survival or cognitive function in 1‐month‐old mice. These results suggest that, during early stages, elevated C3 expression does not exert substantial effects on neural cell populations or fundamental cognitive functions. C1q accumulates at synapses and is associated with synaptic loss in AD mouse models and in post‐mortem brain tissue from individuals with tauopathy. In humans, non‐human primates, and mice, C3 expression increases with age (Li et al. 2013; Wu et al. 2022). Our genetically modified mice exhibited a significant increase in C3 expression, suggesting that, similar to its role during development, C3 activity drives synaptic loss in adulthood. Increased astrocyte secretion of C3, mediated by nuclear factor (NF)‐κB, has been shown to impact neuronal morphology and function (Lian et al. 2015), further highlighting the crucial role of C3 in brain aging.

In the central nervous system, interactions among microglia, astrocytes, and oligodendrocytes are linked to the pathological features of neurodegenerative diseases (Schwartz et al. 1999). With advancing age, dysfunctional mitochondria produce more ROS and activate NF‐κB signaling, which enhances the NOD‐, LRR‐, and pyrin domain‐containing protein 3 (NLRP3) inflammasome, leading to the release of interleukin‐1β in the brain and resulting in age‐related chronic inflammation (Cunningham 2013; Youm et al. 2013). In the present study, C3 transgenic mice substantially more GFAP‐positive cells compared with the control group. The PCR microarray experiment for astrocyte‐associated mRNAs identified CD44, a cell surface receptor that plays a crucial role in cell‐to‐cell interactions, adhesion, and migration, helping cells to sense changes in the tissue microenvironment and respond accordingly (Wu et al. 2014). This finding suggests that C3 influences neuronal function by altering interactions between astrocytes and microglia. Nutritional and metabolic factors may also contribute to cognitive impairment risk. Insulin and IGF1 resistance in the brain are early and common features of AD; caloric restriction has shown neuroprotective effects (Fontana et al. 2010). Moreover, neurons in AD patients exhibit reduced activity concerning the glucose transport proteins GLUT1 and GLUT3, as well as the glycolytic enzyme aldose reductase (Camandola and Mattson 2017). IRS2, a cytoplasmic signaling molecule, mediates the effects of insulin by bridging receptor tyrosine kinases with downstream effectors (Manohar et al. 2020). In our C3 transgenic mice, mRNA levels of IRS2 significantly changed. Additionally, when C3a was added to U251 cells, the mRNA levels of insulin receptors and mitochondria‐related genes in those cells decreased. These findings suggest that complement C3 contributes to learning and memory impairments by modulating insulin signaling in astrocytes.

Astrocytes express active insulin receptors, and astrocytic insulin signaling regulates ATP release from astrocytes, which then modulates the activity of dopaminergic neurons in the midbrain (Chen et al. 2023). Neurotransmitters play a crucial role in learning and memory, serving as chemical messengers that transmit signals between neurons, thereby influencing these processes (Teleanu et al. 2022). In particular, dopamine participates in learning and memory regulation through its receptors, especially in the formation and consolidation of long‐term memory. Dopamine also induces changes in synaptic plasticity and influences the threshold for memory encoding by modulating hippocampal excitability and long‐term potentiation, thus affecting learning and memory. Using targeted metabolomics, we observed significantly lower dopamine levels in C3 transgenic mice than in wild‐type mice. This finding is consistent with previous research showing that the loss of insulin receptors in astrocytes impairs dopaminergic neuronal activity (Cai et al. 2018), which may be a key factor contributing to the observed impairments in learning and memory.

In conclusion, we demonstrated that increased expression of complement C3 leads to impaired learning and memory in mice, as well as synaptic and neuronal loss and astrocytosis. Our findings suggest that C3 is a core molecule associated with age‐related cognitive decline. We also explored the molecular mechanism by which C3 regulates insulin signaling in astrocytes, subsequently activating mitochondrial dysfunction in astrocytes and leading to impaired cognitive function in mice. Additionally, this study utilized antibodies to block complement C3 in mouse brain tissue, successfully reversing learning and memory impairments in SAMP8 mice. These results provide a solid foundation for understanding the role of complement C3 in the development of age‐related cognitive dysfunction and present new evidence to support complement C3 as a potential therapeutic target for cognitive disorders.

## Author Contributions

J.L., B.C., N.W., and J.Z. conceived and designed the project; M.D., X.W., Y.W., Y.L., F.X., X.M., and K.M. conducted animal and in vitro experiments; M.D., Y.W., and X.W. analyzed data; M.D. and Y.W. wrote the manuscript.

## Ethics Statement

All experiments were performed in accordance with protocols approved by the Committee of Experimental Animal Administration of Capital Medical University, China (No. AEEI‐2021‐228).

## Conflicts of Interest

The authors declare no conflicts of interest.

## Supporting information


Data S1.

## Data Availability

Data generated or analyzed during this study are available from the corresponding author upon reasonable request.
