# Supplementary material for: Age‐Related Complement C3 Drives Memory Impairments and Associated Neuropathologies in a Mouse Model
Source: Aging Cell. 2025 Jun 20;24(9):e70145. doi: 10.1111/acel.70145 (PMC12419844; doi:10.1111/acel.70145)
Supplement: Supplementary file 1 — Data S1. [file ACEL-24-e70145-s001.docx]

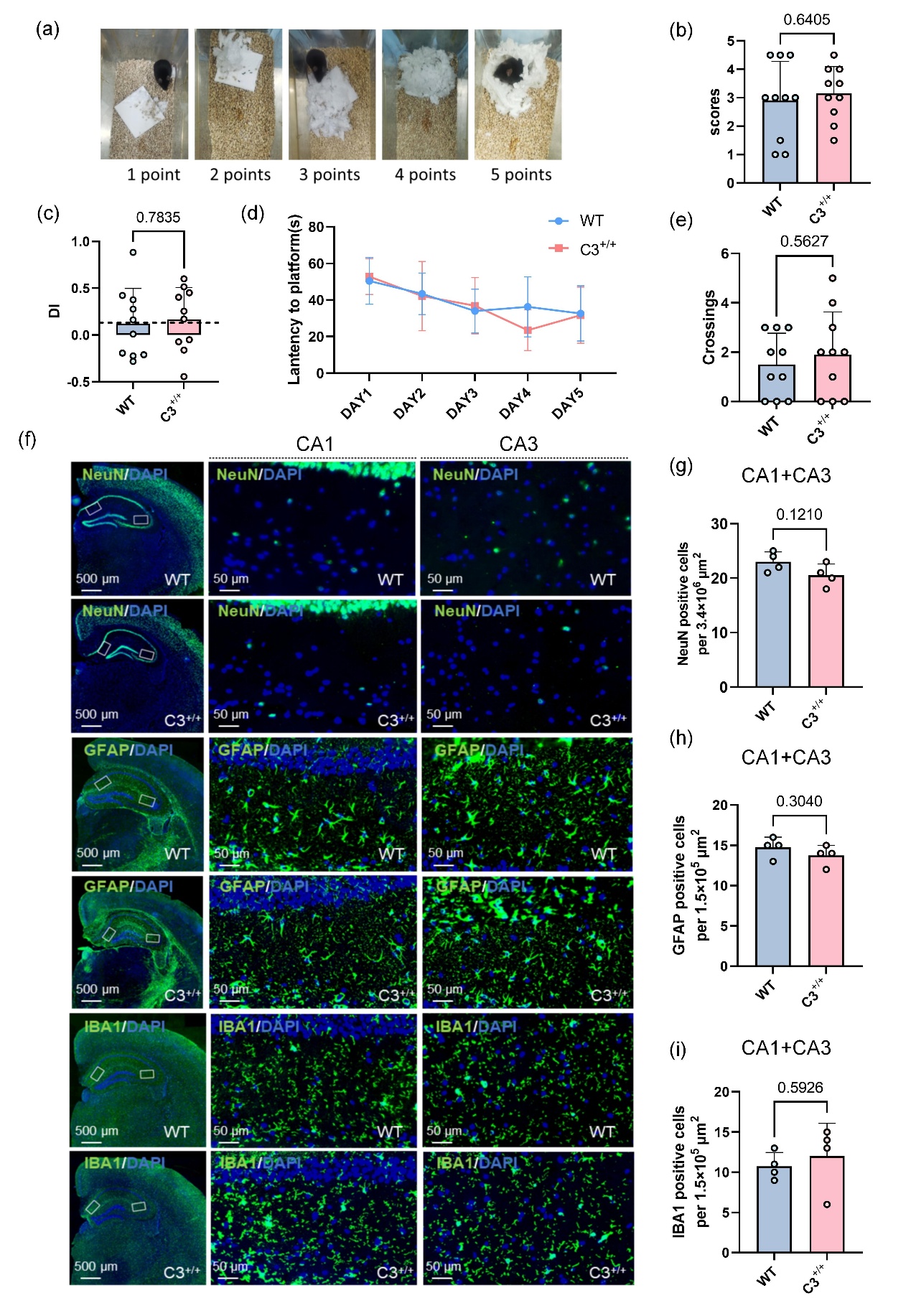


**Fig S1:** **Absence of learning and memory impairment in 3-month-old** **C3 transgenic mice.**

(a) Quantitative scoring criteria for nesting behavior in C3 transgenic mice: a score of 1 was assigned when more than 90% of the square compressed cotton sheets remained intact; 2, when 50%-90% remained intact; 3, when 50%-90% were shredded and no recognizable nest had formed; 4, when more than 90% were shredded and a recognizable but relatively flat nest was formed; and 5, when more than 90% were shredded and a nearly complete nest was observed. (b) Analysis of mouse nesting behavior. (c) Discrimination index in the novel object recognition test for 3-month-old C3 transgenic and age-matched wild-type mice (mean values from n=10 [WT] and n=10 [C3+/+] per group; unpaired Student's t-test). (d) Latency during water maze training in 3-month-old C3 transgenic and wild-type mice (n=10 per group; two-way ANOVA). (e) Number of platform crossings in the water maze test (unpaired Student's t-test). All data are presented as mean ± standard deviation (SD). (f) Representative immunofluorescence images of hippocampal sections from 1-month-old C3 transgenic mice and age-matched wild-type (WT) mice, showing staining for NeuN (green) to label neurons, GFAP (green) to label astrocytes, and IBA1 (green) to label microglia. (g) Quantification of NeuN-positive cells in the hippocampus of WT (n=4) and C3 transgenic mice (n=4) (unpaired Student’s t-test). (h-i) Quantification of GFAP-positive and IBA1-positive cells in the hippocampus of WT (n=4) and C3 transgenic mice (n=4) (unpaired Student’s t-test). Scale bars represent 500 μm and 50 μm. All data are presented as mean ± standard deviation (SD). Individual data points represent biological replicates.


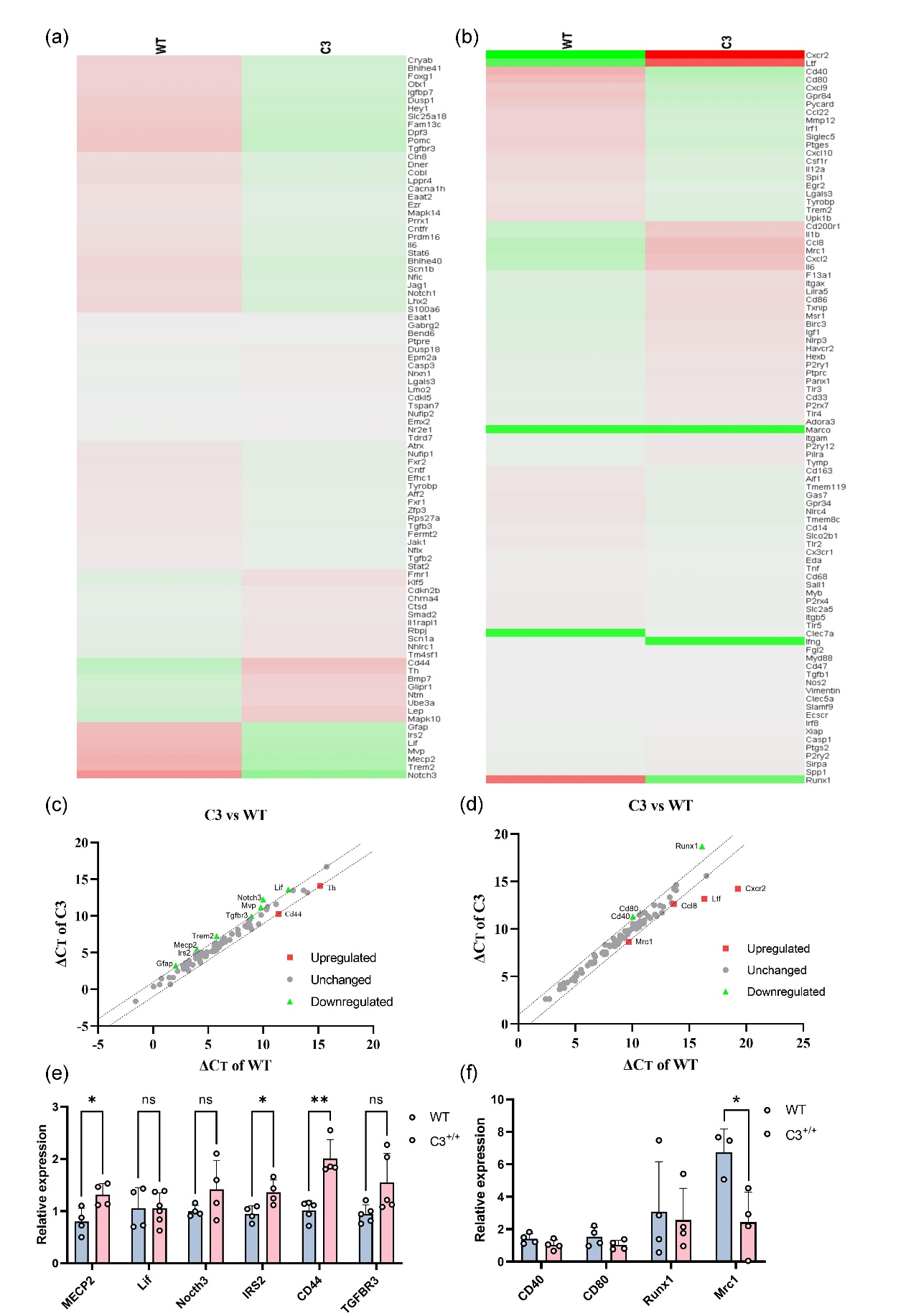


**Fig S2: Molecular analysis of microglial differences.** (a-b) Heatmap of molecular expression in astrocytes from qPCR astrocytes and microarrays of brain in C3 transgenic mice (7-8 months old [both C3 transgenic mice and WT mice]) and WT mice (n=1 per group, WT and C3^+/+^). (c-d) Differential molecular analysis of astrocytes and microarrays shown in panel a and panel b. (e-f) Validation of significant mRNAs identified through qPCR microarray analysis in astrocytes (n=4 per group, WT and C3^+/+^; unpaired Student’s t-test).


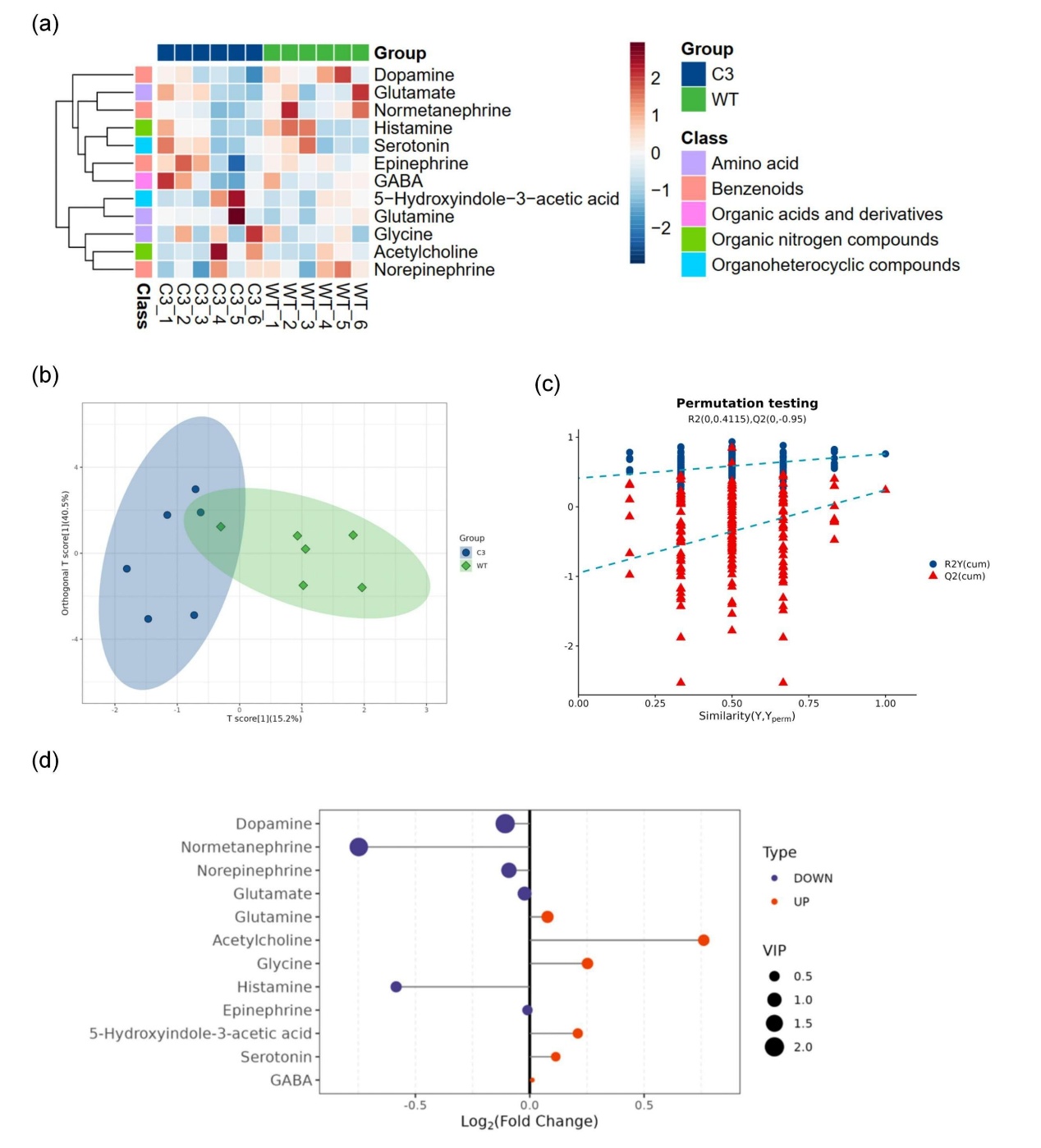


**Fig S3: Neurotransmitter characteristics in WT and C3 transgenic mice.** (a) Heatmap showing neurotransmitter levels in the brain. (b) Orthogonal partial least squares discriminant analysis (OPLS-DA) of 6-month-old WT (n=6) and C3 transgenic mice (n=6). (c) Permutation test for the OPLS-DA analysis. (d) Expression levels of neurotransmitter metabolites in the two groups, with dot size indicating variable importance in projection (VIP) values; larger dots represent higher VIP values, signifying greater variable importance.

**Table S1: Gene Symbol Primer direction Primer sequence**

|  | **Gene Symbol** | **Primer sequence (5**′**- 3**′**)** |
| --- | --- | --- |
| 1 | *IR* | F: AAATGCAGGAACTCTCGGAAGCCT  R: ACCTTCGAGGATTTGGCAGACCTT |
| 2 | *mtND1* | F: TGCCAGCCTGACCCATAGCC  R: ATGGGCCGGCTGGGTATTCT |
| 3 | *mtCytB* | F: ACCAATCTCCCAAACCATCA  R: TCCAGAGACTTGGGGATCTAAC |
| 4 | *mtATP6* | F: CAGTCCCCTCCCTAGGACTT  R: TCAGAGCATTGGCCATAGAA |
| 5 | *RUNX1* | F: CCGAGAACCCCGAAGACATC  R: GGCTGAGGGTTAAAGGCAGT |
| 6 | *CD80* | F: ACCCCCAACATAACTGAGTCT  R: TTCCAACCAAGAGAAGCGAGG |
| 7 | *CD40* | F: ATGCCACCCATGTGACTCAG  R: GGTGCCCTCCTTCTTAACCC |
| 8 | *MRC1* | F: CTCTGTTCAGCTATTGGACGC  R: CGGAATTTCTGGGATTCAGCTTC |
| 9 | *MECP2* | F: TATTTGATCAATCCCCAGGGAAA  R: CTCCCTCTCCCAGTTACCGT |
| 10 | *LIF* | F: CGGAAGCGAGAATGGATTAAGGA  R: GACCGAGATTCCAGGACTTCAAC |
| 11 | *NOCTH3* | F: GATCAAGACATTGACGACTGTGAC  R: GTCGAGGCAAGAACAGGAAAAG |
| 12 | *IRS2* | F: CGAGTCAATAGCGGAGACCC  R: CCCCTGAGACCCTACGGTAA |
| 13 | *CD44* | F: ACTTTGCCTCTTGCAGTTGAG  R: TTTCTCCACATGGAATACACCTG |
| 14 | *TGFBR3* | F: GGTGTGAACTGTCACCGATCA  R: GTTTAGGATGTGAACCTCCCTTG |
| 15 | *β-actin* | F: TCTGGCACCACACCTTCTAC  R: ACGACCAGAGGCATACAGG |
